# Uniform Polishing Method of Spherical Lens Based on Material Removal Model of High-Speed Polishing Procedure

**DOI:** 10.3390/mi11100938

**Published:** 2020-10-15

**Authors:** Hao Zhang, Peng Wang, Zexiao Li, Yi Shen, Xiaodong Zhang

**Affiliations:** 1 State Key Laboratory of Precision Measuring Technology & Instruments, Centre of Micro Nano Manufacturing Technology, Tianjin University, Tianjin 300072, China; zhanghao1985@tju.edu.cn (H.Z.); lzxfade@163.com (Z.L.); 2 Tianjin Jinhang Institute of Technical Physics, Tianjin 300308, China; wpeng605@126.com (P.W.); sylgg_06@126.com (Y.S.)

**Keywords:** Preston equation, high-speed polishing, large-size silicon lens, uniform polishing method

## Abstract

Although the high-speed polishing technology has been widely applied to obtain an ultra-smooth surface in the field of spherical optical manufacture, it is still mainly used in small-size or easily polished lenses. In the infrared optical system, large-size silicon lenses are often used to increase the luminous flux. As is known, the material is hard-polished, it is time-consuming to reduce the surface roughness by iterative polishing and it is difficult to avoid the form accuracy getting worse. To produce an ultra-smooth surface efficiently without destroying the figure, a scientific understanding of material removal in the high-speed polishing process is necessary, which would lead to the process being more deterministic. In this paper, a mathematical model of material removal is developed based on the classic Preston equation. The predicted results of the proposed model show good agreement with the experimental data. Further, a method to achieve uniform polishing can be addressed with a systematic analysis of the key factors affecting material removal and their contribution to spatial non-uniform removal. Finally, the experimental results indicate that the surface roughness of hard-polished spherical optics can be improved efficiently using the uniform polishing method without the surface figure being destroyed.

## 1. Introduction

It is universally recognized that the accuracy of a spherical optical lens is easy to measure and spherical polishing machines are inexpensive compared with aspherical polishing machines. As a result, spherical lenses have been applied widely and become a fundamental sort of element in the optical industry [1]. Like aspherical optics, the fabrication technology of the spherical lens with sub-micrometer form accuracy and nanometer surface efficiency is always attractive, especially in the field of mass production of difficult-to-polish materials [2]. Early in the 1960s, significant progress was achieved by introducing high-speed polishing to meet the demand for massive industrialized production [3,4]. Different from the classical method, the polishing tool is covered by a polyurethane pad which is a thermosetting material and its rotation speed is much higher. In effect, a reduction in the polishing time by a factor of 5 to 10 can be achieved. In the past decades of development, the high-speed polishing technology has become increasingly popular because of its cost-effectiveness, process consistency and short production time. Generally, it only takes a few seconds to finish polishing for small-size glass lenses, which can avoid the surface figure getting worse. However, in some kinds of infrared optical systems, the materials are often hard-polished such as silicon and sometimes the size is large in order to increase the luminous flux. Compared with small-size glass lenses, the polishing time of the large-size silicon lens is much longer (several hours typically). It is inefficient and cumbersome to reduce the surface roughness without destroying the surface figure by iterative adjustments based on craftsman-like skill. Hence, developing a scientific understanding of the material removal of high-speed polishing technology would lead to a more deterministic process, allowing the manufacture of the large-size infrared lens, which is made of hard-polished materials, to be performed in a more efficient, less iterative manner.

**Figure 1:**
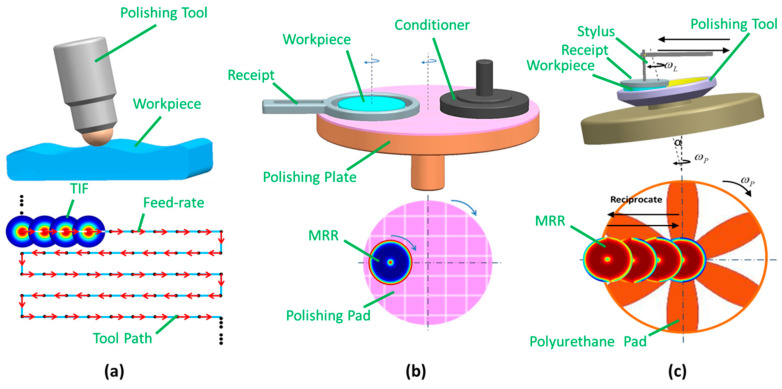
Illustration of different polishing processes: (**a**) bonnet polishing; (**b**) chemical mechanical polishing (CMP); (**c**) high-speed polishing.

In the polishing process, material removal can be described by the Preston equation [5]

(1) $$\frac{d\Gamma }{dt}=k_{0}p\left(x,y\right)v\left(x,y\right)$$

where *d*Г/*dt* is the material removal rate, *p*(*x,y*) is the instantaneous pressure distribution and *v*(*x,y*) is the instantaneous relative velocity field of the workpiece surface. The molecular-level effects are described macroscopically by the Preston coefficient *k_0_* which is often regarded as an invariant constant in a process [5]. This equation shows that the key issue of the calculation of the material removal rate is establishing an effective mathematical model of pressure and relative velocity distribution on the workpiece surface. Obviously, the research methodologies vary according to the polishing technology which can be divided into two types based on the size of the polishing tool, i.e., sub-aperture type and full-aperture type. In a sub-aperture polishing process, the polishing tool is much smaller than the workpiece (see Figure 1a) and material removal distribution is achieved by controlling the feed rate (or tool path) on the workpiece surface [6,7]. The program is typically computed according to the tool influence function (TIF) [8] and the surface form error [9,10]. This is mainly used in aspherical optical manufacture. For flat or spherical optics, full-aperture-type polishing technology is widely used. Different from the sub-aperture type, the polishing tool is larger than the workpiece (see Figure 1b,c) and material removal distribution is obtained by controlling a series of processing parameters (rotation speed, load pressure and so on). As shown in Figure 1b, the relative position between the workpiece and the polishing tool is fixed so the material removal rate (MRR) can be treated as “static” for the flat polishing process. However, for spherical optics in high-speed polishing technology (see Figure 1c), due to the reciprocating motion between the tool and workpiece, the shape of the material removal distribution changes continuously with the process time. In other words, the mathematical model of material removal for high-speed polishing technology, which is typically used in spherical optics, is quite different from the flat polishing process.

During recent decades, the study on the establishment of deterministic kinematic models is finding overwhelming applications in different kinds of the polishing process. For sub-aperture polishing, it has attracted a lot of attention. Jones proposed the theoretical TIF and optimized the process by computer simulations [11]. Dai et al. established a calibrated and predictive TIF model of magnetorheological finishing [12]. Lin developed an analytical model to describe the relationship between polishing parameters and TIF [13]. Undoubtedly, the study of deterministic kinematic models is an essential issue for improving surface accuracy in some kinds of sub-aperture polishing. For full-aperture polishing, Martinez et al. studied the motion of the working tool in the flat polishing process [14]. Dein Shaw and Jassy Chang studied the optimization of pressure distribution in chemical mechanical polishing (CMP) [15]. Onemoon Chang et al. presented a mathematical model for the general conditioning process with oscillation in CMP [16]. Sun et al. proposed a computational model of a polishing process on small-radius spherical surfaces by the finite element analysis (FEA) method [17]. However, experimental results are not so consistent with the theoretical values, which is because of the approximation error of the numerical computational method. Moreover, the analysis procedure is very time-consuming, which may become a major obstacle in practice. Based on the literature study, it is found that much research has been performed on the study of the establishment of deterministic kinematic models for flat polishing (such as CMP) [14,15,16,18,19,20], but few studies have been found on spherical polishing [17], especially about the high-speed polishing process. This leads to the fact that the high-speed polishing technology is still limited to small-size or easily polished lenses.

**Figure 2:**
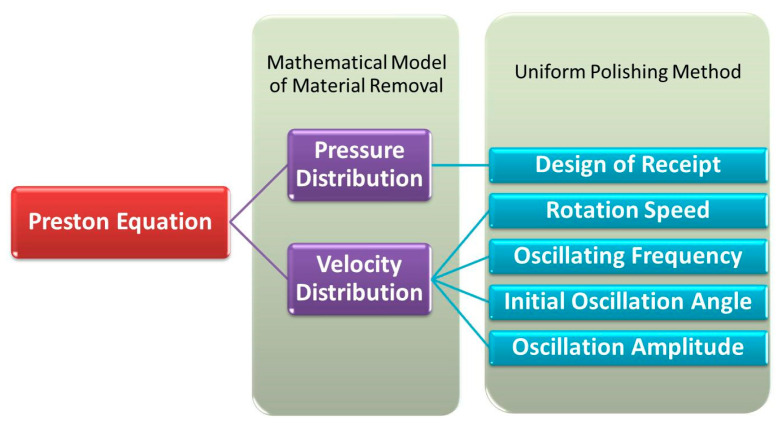
Framework of this paper.

In this paper, a mathematical model of material removal is developed for the high-speed polishing of spherical optical elements based on the classic Preston equation. The distribution of relative velocity on the surface is calculated by the proposed kinematics model of the high-speed polishing process, and pressure on the surface is analyzed according to the theory of elasticity. Hence, the distribution of material removal during a polishing process can be predicted. Based on the mathematical model, the effects on non-uniform spatial material removal are simulated, and the effectiveness of the simulation is verified by experiments. Consequently, a uniform polishing method of the spherical optical element is demonstrated. It is shown that the surface roughness can be reduced without destroying the figure less iteratively. The framework of this paper is shown in Figure 2.

## 2. Modeling of High-Speed Polishing 

Figure 1c shows the schematic of the high-speed polishing equipment. A polishing tool with a polyurethane pad is mounted on the spindle. The tilt angle *α* is calculated by

(2) $$\alpha =\frac{\alpha_{\mathrm{tool}}-\alpha_{\mathrm{len}}}{2}$$

where *α*_tool_ and *α*_len_ are the central angle of the polishing tool and the workpiece, respectively. The reciprocating rectilinear movement of the workpiece is driven by the stylus.

According to the Preston equation (Equation (1)), material removal is the function of pressure *p*(*x, y*), relative velocity *v*(*x, y*), processing time *t* and the coefficient *k*_0_. As mentioned above, *k_0_* is always considered as an invariant constant, so the key issue of establishing a mathematical model of material removal is expressing the pressure and relative velocity at a given point on the polishing surface in an analytical way.

### 2.1. Pressure Distribution

**Figure 3:**
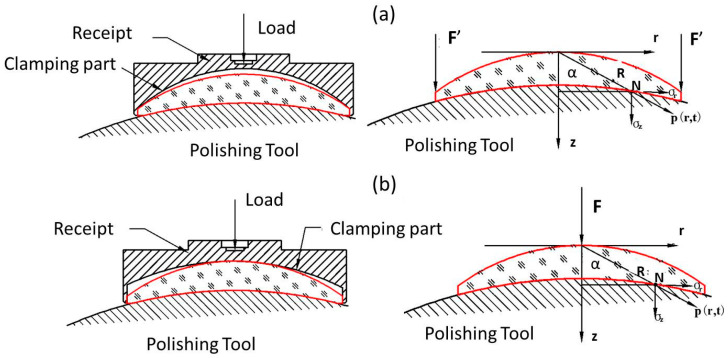
Relationship between receipt and pressure distribution: (**a**) center contact; (**b**) edge contact.

It is obvious that the clamping part of the receipt has an essential influence on the polishing pressure distribution (Figure 3). Ideally, the radius of the clamping part is strictly equal to the contact surface of the workpiece. However, a machining error often occurs, unavoidably. It means that the applying position of load *F* is quite different, as shown in Figure 3.

Considering that the radius of the clamping part is larger, *F* is applied at the center of the contact surface, as shown in Figure 3a. A polar coordinate system is defined and Point O is set as the origin. The instantaneous pressure at the given Point N **p**(*r, t*) can be expressed as

(3) $$p\left(r,t\right)=\sigma_{r}\left(r,t\right)+\sigma_{z}\left(r,t\right)$$

According to the Boussinesq solution in the theory of elasticity [21], both **σ***_r_*(*r, t*) and **σ***_z_*(*r, t*) are symmetric about the *z*-axis.

(4) $$\left\{ \begin{array}{l} \sigma_{r}=\frac{1}{2\pi }F\left[\frac{\left(1-2\nu \right)}{R\left(R+z\right)}-\frac{3r^{2}z}{R^{5}}\right]\\ \sigma_{z}=-\frac{3}{2\pi }F\frac{z^{3}}{R^{5}} \end{array}\right.$$

The instantaneous pressure field **p**(*r, t*) is calculated by substituting Equation (4) into Equation (3). Contrariwise, when the radius of the clamping part is less than the contact surface, *F* is distributed on the edge, as shown in Figure 3b. 

**Figure 4:**
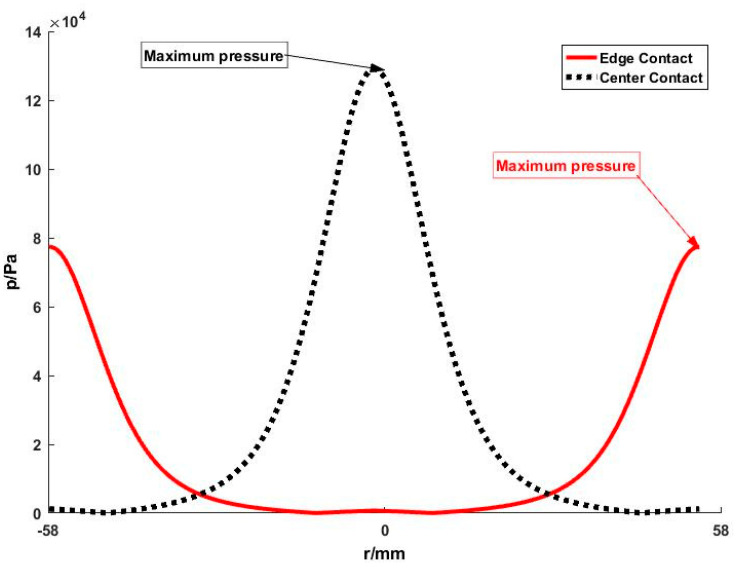
Pressure distribution of different receipts: the maximum pressure occurs at the contact position and it is much lower for the edge contact compared with the center contact.

The influence of two different receipts on pressure distribution is compared in Figure 4. It can be seen that the contact position has a significant effect on pressure distribution, which leads to a non-uniformed material removal rate.

### 2.2. Analysis of Relative Velocity

The relative velocity **v** of a given point N on the polishing surface is shown as

(5) $$v=v_{P}-v_{L}+v_{A}$$

where **v***_p_* is the rotation velocity of the pad, **v***_L_* is the rotation velocity of the workpiece and **v***_A_* is the translation velocity of the workpiece driven by the stylus. **v***_A_* is the cross product of the motion of the stylus (which can be calculated by the oscillation frequency *f* directly) and the radius of the polishing surface.

Concerning the workpiece rotation, the rotational torque is produced by friction and the supporting force from the stylus. According to the law of inertia, *ΣM* = *Ia*, where *I* is the moment of inertia, and *a* is angular acceleration. For a given Point *N*(*r, θ*), d*M = μp*(*x, y*)*π*[(*r + dr*)^2^ *− r*^2^], so

(6) $$\sum M=\int_{0}^{R_{2}}2\mu p\left(x,y\right)\pi rdr=m_{2}{\left(\frac{D}{2}\right)}^{2}a$$

where *μ* is the coefficient of friction, *m_2_* is the mass of the workpiece and *D* is the diameter of the workpiece. The angular velocity of the workpiece ***ω****_L_* can be calculated by time integration of *a* according to Equation (6). The coefficient of friction *μ* can be determined by fitting equation $\mu =0.45\omega_{P}^{-0.971}$, which is suitable in the polyurethane-type polishing process.

In a word, the angular velocity of the workpiece *ω_L_* is shown as

(7) $$\left\{ \begin{array}{l} \begin{array}{l} \mu =0.45\omega_{p}^{-0.971}\\ 2\int_{0}^{R_{2}}\mu p\left(x,y\right)r\pi dr=m_{2}{\left(\frac{D}{2}\right)}^{2}a\\ \omega_{L}=\int_{0}^{t}adr \end{array}\}if\;\omega_{L}<\omega_{P}\\ \omega_{L}=\omega_{P} \end{array}\right.$$

As a result, **v***_L_* at a given point is determined by **v***_L_* = **ω***_L_* × **r**.

**Figure 5:**
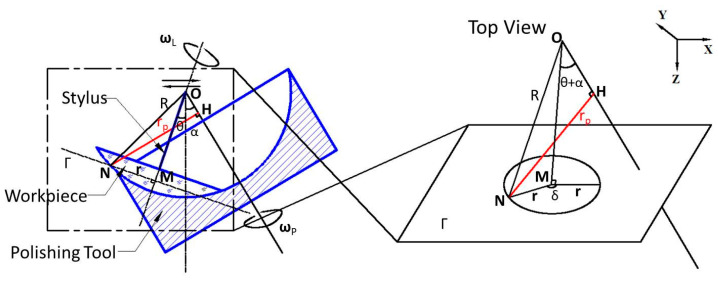
Geometric of the process.

The rotation velocity of pad **v**_p_ at point N is determined by **v***_P_* = **ω***_p_* × **r***_p_*, where **ω***_p_* is the angular velocity of the pad, **r***_p_* is the distance between point N and the rotary axis OH, as shown in Figure 5, *NH* = **r***_p_*, and plane *Г* is perpendicular to the rotary axis OH and goes through point N. A Cartesian coordinate system is defined and the center of sphere point O is set as the origin. The rotary axis of the pad OH is expressed as

(8) $$\left\{ \begin{array}{l} x=k\sin\left(\alpha +\theta \right)\\ y=0\\ z=k\cos\left(\alpha +\theta \right) \end{array}\right.\;(k\;\mathrm{is}\;a\;\mathrm{parameter})$$

and the coordinate of point N is $\left(rcos\delta ,rsin\delta ,\sqrt{R^{2}-r^{2}}\right)$. Accordingly,

(9) $$r_{P}=\left|NH\right|=\min\sqrt{{\left(r\cos\delta -k\sin\left(\alpha +\theta \right)\right)}^{2}+{\left(r\sin\delta \right)}^{2}+{\left(\sqrt{R^{2}-r^{2}}-k\cos\left(\alpha +\theta \right)\right)}^{2}}$$

(10) $$r_{P}=\sqrt{R^{2}-{\left[r\sin\left(\alpha +\theta \right)\cos\delta +\sqrt{R^{2}-r^{2}}\cos\left(\alpha +\theta \right)\right]}^{2}}$$

**Figure 6:**
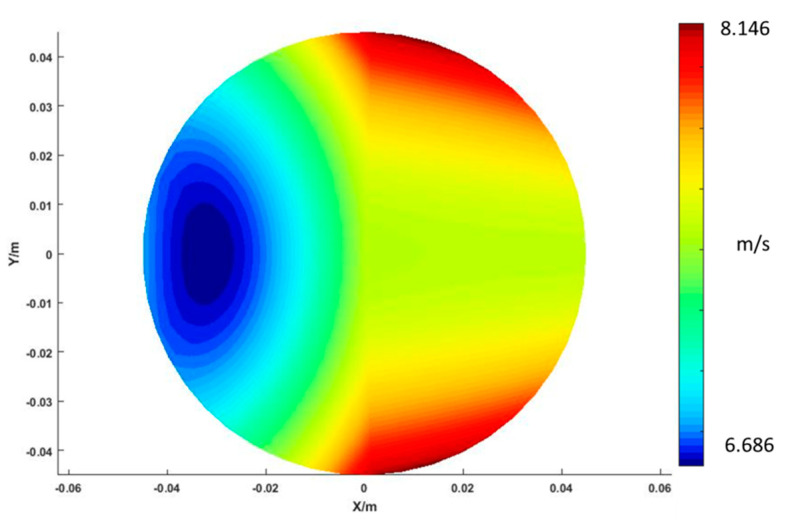
Calculation results of the relative velocity distribution for polishing parameters at α = 9°, **ω***_p_* = 500 RPM, *D* = 90 mm, *R* = 175 mm, *f* = 10 Hz, *θ* = 24°.

It should be noted that *r_p_* is a function of time. As a result, the relative velocity of a given point N can be calculated by Equation (5). Figure 6 displays an example of the calculation results of the relative velocity distribution for polishing parameters at *α* = 9°, ω*_p_* = 500 RPM, *D* = 90 mm, *R* = 175 mm, *f* = 10 Hz, *θ* = 24°.

## 3. Experimental Study

### 3.1. Setup of Experiments

**Figure 7:**
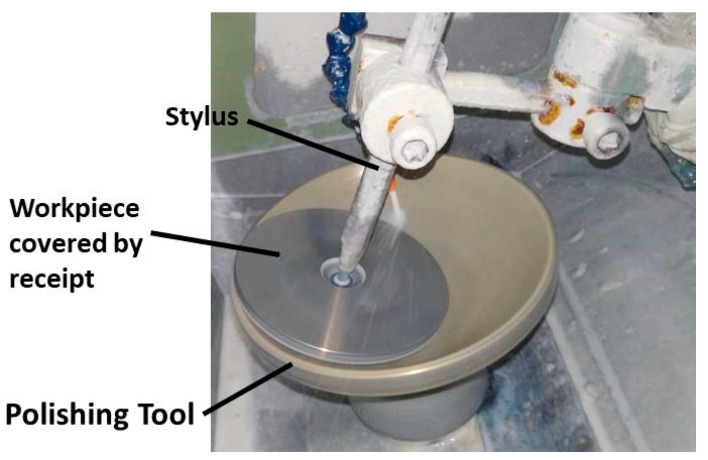
High-speed polishing experiments.

The effectiveness and practicability of the mathematical model are demonstrated with a group of experiments that are carried out on a KJ-4 polisher (see Figure 7) provided by Kwangjin Precision Co. Ltd (Pohang-si, Korea). A convex silicon spherical surface with a radius of 175 mm and a diameter of 90 mm is taken as a sample. A semi-rigid polishing tool which consists of a GR-35 polyurethane layer (manufactured by Universal Photonics Inc., Central Islip, NY, USA) and an aluminum alloy bowl-like body (radius 175 mm, diameter 150 mm) is utilized in the study. Before the experiments, the polyurethane pad is dressed using a ball-like tool covered by diamond pellets. The polishing slurry consists of SiO_2_ abrasives with an average size of 0.17 μm and deionized water, the concentrate is 20 vol%.

### 3.2. Experimental Design and Conditions

As shown in Table 1, there are three tests in this study to investigate the effectiveness of the mathematical model of material removal systematically. The material and dimension of the specimens have been mentioned before. In test A, the initial form errors (the root of the mean square value, RMS) of all the specimens which are finely polished by pitch are less than 10 nm. Furthermore, a group of polishing experiments under different conditions is implemented. The experiment parameters are listed in Table 2. The polishing process continued for two hours to investigate the material removal. In test B and test C, six specimens are initially fine-ground by diamond pellets, and three of them are used in test B. The surface figure is improved by adjusting the polishing parameters according to skillful operators’ experience independently and is measured by a laser interferometer (GPI. ZYGO) every 30 min. Other specimens are used in test C. Different from test B (empirical polishing method), the processing parameters are determined by the material removal model proposed in Section 2. Moreover, the surface figure is measured every 30 min.

## 4. Results and Discussion

### 4.1. Test A: Validation of the Mathematical Model

**Figure 8:**
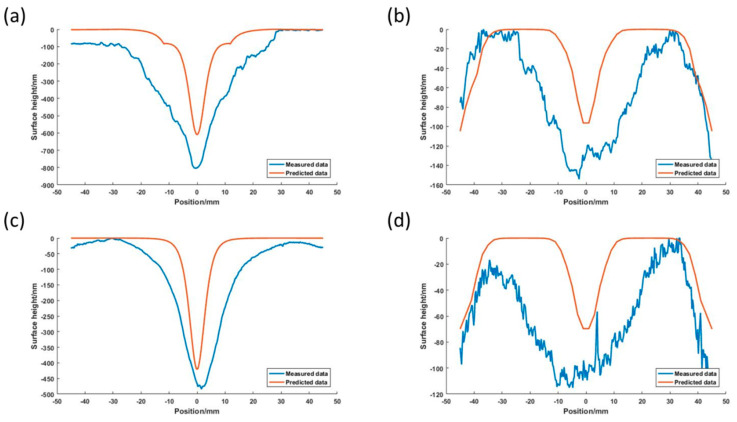
Comparison of predicted and experimental results: (**a**) is Trial No.1; (**b**) is Trial No.2; (**c**) is Trial No.3; and (**d**) is Trial No.4.

**Figure 9:**
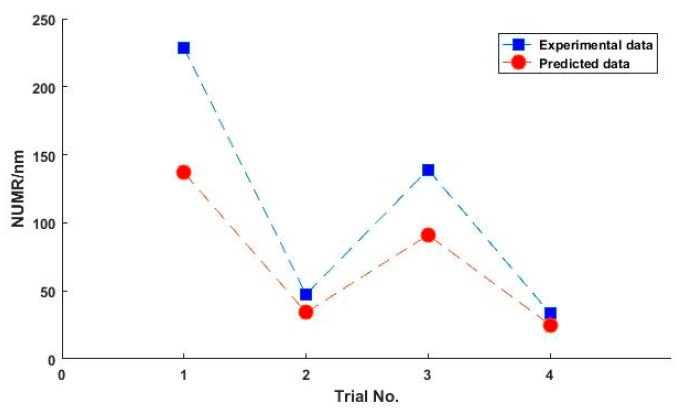
Comparison of the predicted and the experimental value of *NUMR.*

A series of simulations are conducted under the same conditions as shown in Table 2. Figure 8 shows the material removal profile measured by the ZYGO Interferometer and the predicted results by the mathematical model proposed in this paper. It can be seen that there is a high value of material removal at the center in Trial No.1 and Trial No.3. In comparison, the material removal is uniform in Trial No.2 and Trial No.4. The uniformities of material removal are quite different in the different trials. This indicates the distribution of material removal on the polishing surface which is the essential issue for improving the form accuracy depending on the processing parameters. So, it is meaningful to develop a scientific understanding of the relationship between processing parameters and the distribution of material removal through an effective mathematical model. To validate the effectiveness of the proposed model in the prediction of the distribution of material removal, the non-uniformity of the distribution of material removal is described by the value of *NUMR* defined as

(11) $$NUMR=\sqrt{\sum_{i=1}^{n}{\left(\Gamma_{i}-\bar{\Gamma }\right)}^{2}}$$

where Г*_i_* is material removal at point *i*, $\bar{\Gamma }$ is the average material removal on the polished surface and *n* is the number of discrete points. A lower *NUMR* indicates more uniform material removal. Figure 9 shows a comparison of the predicted and the experimental value of *NUMR* in test A. The predicted *NUMR* shows a consistent changing trend with the *NUMR* of the experimental data. Moreover, the surface shape of the predicted results shows a good correlation with the experimental data, although the height deviation is in a range of tens of to two hundred nanometers, as shown in Figure 8. Considering the mathematical model, the deviation may be caused by a series of simplifications. Obviously, the thickness of the adhesive layer of the polyurethane pad, slurry distribution on the polishing surface and the environmental temperature are not taken into account. However, the experimental results in this test show that the correlation between distributions of material removal and processing parameters can be obtained through the mathematical model proposed in this paper.

Furthermore, it is interesting to note that the contact position between the receipt and workpiece has a significant effect on material removal. Obviously, center-contact results in a high value of the material removal rate at the center of the polishing surface whatever the parameters are. It means that the surface accuracy is difficult to improve by adjustment of the process parameters in this case. However, edge-contact leads to material removal that is more controllable because the pressure distribution is more uniform as illustrated above (see Figure 4). So, it is strongly advised that the receipt should be designed in an edge-contact style.

### 4.2. Test B: Empirical Polishing

**Figure 10:**
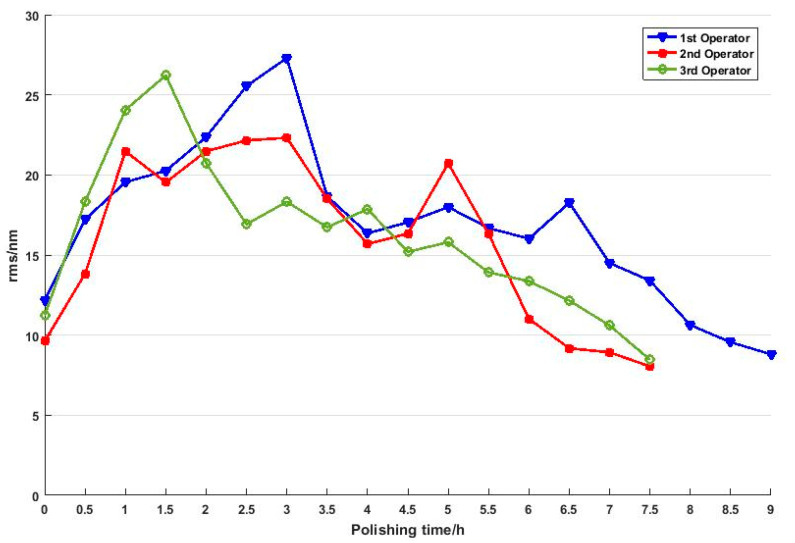
Measurement results of test B.

**Figure 11:**
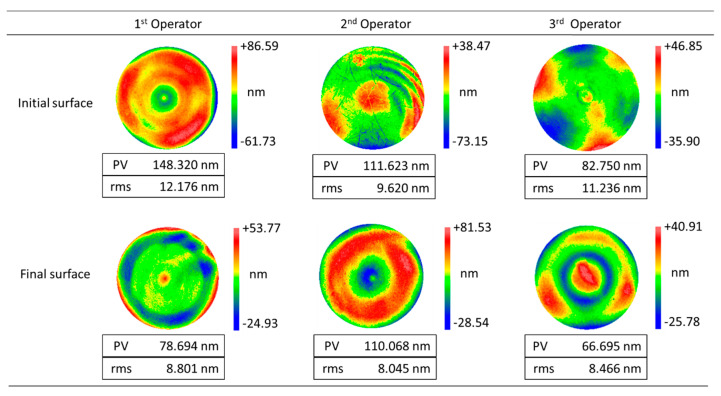
Profile of the initial and final surface form errors in test B.

The polishing time and the corresponding measurement results of surface form errors (RMS value) are shown in Figure 10. The RMS value increases at the beginning and then decreases in a fluctuating way. This is a general phenomenon in empirical polishing because operators usually specify processing parameters according to his/her experience at first and improve the surface figure by iterative measurements and adjustments. This leads to the fact that the empirical polishing process based on craftsman-like skill is inefficient and unpredictable. Furthermore, it should be noted that the profile of the final surface form errors is quite different from the initial one, as shown in Figure 11. The measurement results indicate material removal is non-uniform during the polishing process.

**Figure 12:**
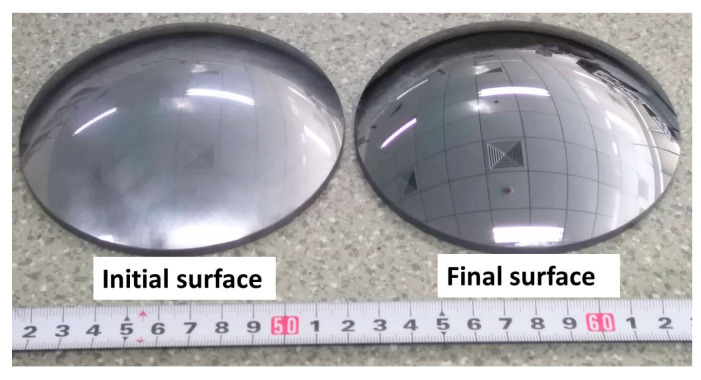
A picture of the initial surface (**left**) which is ground by diamond pellets and the final surface (**right**) in test B.

More importantly, it is known that a high-precision initial form can be obtained by grinding ordinarily, although the surface is far from mirror-like (see Figure 12, the left one). Ideally, the material removal in the polishing process could be performed uniformly. It means the surface roughness can be reduced gently. So, improving the uniform distribution of material removal in the polishing process is an efficient method to obtain high-precision optical surfaces.

Based on the mathematical model proposed in this paper, the effects of processing parameters on non-uniform spatial material removal can be simulated. In general, polishing parameters contain the rotation speed of the polishing tool, oscillating frequency, initial oscillation angle and oscillation amplitude. So, the non-uniform material removal should be mitigated by adjusting these parameters.

**Figure 13:**
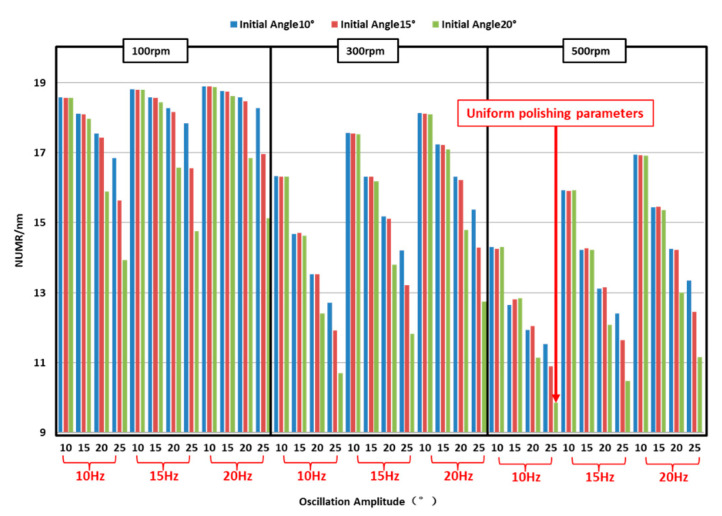
Non-uniformity of material removal for different parameters, where the group of parameters which can achieve uniform polishing is marked.

A series of processing parameters are listed in Table 3. The non-uniformity of material removal is described by the value of *NUMR*, as illustrated above (Equation (11)). Systematical comparisons of the parameters are shown in Figure 13. It can be inferred that *NUMR* is reduced by increasing the rotation speed of the polishing tool, the oscillation amplitude of the stylus and the initial oscillation angle whilst decreasing the oscillating frequency. Consequently, the processing parameters which can achieve uniform polishing are listed in Table 4.

### 4.3. Test C: Uniform Polishing

**Figure 14:**
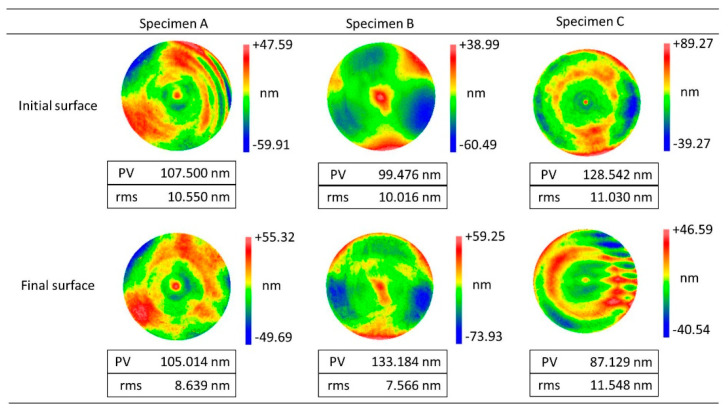
Profile of the initial and final surface form errors in test C.

The profile of initial surface form errors and the polished ones using the uniform polishing parameters listed in Table 4 are shown in Figure 14. Compared with test B (see Figure 11), it is obvious that the shape of the initial and final surface form errors is similar in test C. It means uniformity of material removal is achieved by optimizing the polishing parameters.

**Figure 15:**
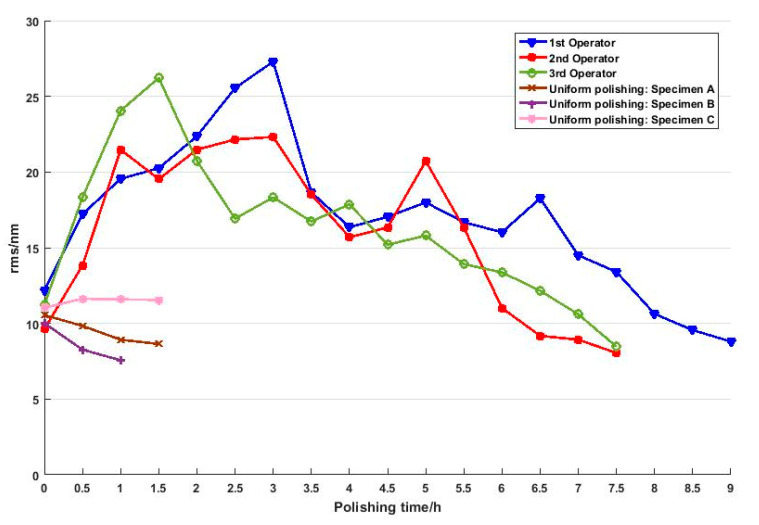
A comparison of experimental results of empirical polishing (test B) and uniform polishing (test C).

Figure 15 shows the measurement results and polishing time in test C and test B. It can be confirmed that high-precision polishing is achieved in fewer iterations using a single set of polishing configurations without the need for in-process surface figure metrology and adjustments in test C. This is because the influence factors on non-uniform spatial material removal are reduced by optimizing polishing configurations based on the mathematical model. In effect, surface roughness declines gradually without destroying the surface figure. As a result, the efficiency is improved dramatically, and the manufacture of large-size hard-polished optics can be performed in a more deterministic, less iterative manner.

## 5. Conclusions

This paper presents a mathematical model of material removal characteristics in high-speed polishing for spherical optical manufacture. The model is developed by making use of the concepts of kinematics, Preston equations and time dependence analysis. Based on this model, the distribution of material removal on the polishing surface can be predicted and the major findings are summarized as follows.

1. Surface accuracy is difficult to improve if the workpiece contacts with the receipt at the center, while edge-contact leads to material removal that is more controllable.
2. In general, the non-uniform material removal should be mitigated by optimization of the rotation speed of the polishing tool, oscillation amplitude of the stylus, initial oscillation angle and oscillating frequency.
3. With a systematic understanding of the key factors affecting material removal and their contribution to spatial non-uniform removal, a method to achieve the goal of uniform polishing is described. Experimental results show that this technique leads to processes that are more deterministic and allows large-size or hard-polished spherical optical manufacture to be performed more efficiently.

## Figures and Tables

**Table 1:** Arrangement of experiments.

Test A: Validation of the Mathematical Model	Test B: Empirical Polishing	Test C: Uniform Polishing
Specimens are initialized at a high-precision level. Polishing tests by different processing parameters. Comparison of the predicted results and the experimental results.	Choose three skillful operators. Polish three specimens independently and improve surface figures by adjusting parameters according to his/her experience. Measure the surface figure every 30 min.	Polish three specimens using the uniform polishing method. Measure the surface figure every 30 min.

**Table 2:** Process parameters of test A and simulations.

Trial No.	Rotation Speed (RPM)	Oscillating Frequency (Hz)	Initial Oscillation Angle (degree)	Oscillation Amplitude (degree)	Load (N)	Style of Receipt
1	500	10	20	15	90	Center-contact
2	500	10	20	15	90	Edge-contact
3	300	10	25	15	180	Center-contact
4	300	10	25	15	180	Edge-contact

**Table 3:** Processing parameters.

Rotation Speed (rpm)	Oscillating Frequency (Hz)	Initial Oscillation Angle (degree)	Oscillation Amplitude (degree)
100	10	10	10
300	15	15	15
500	20	20	20
-	-	-	25

**Table 4:** Uniform polishing parameters.

Rotation Speed	Oscillating Frequency	Initial Oscillation Angle	Oscillation Amplitude	Style of Receipt
500 rpm	10 Hz	20°	25°	Edge-contact
